# Initial Coin Offerings: Risk or Opportunity?

**DOI:** 10.3389/frai.2020.00018

**Published:** 2020-04-16

**Authors:** Anca Mirela Toma, Paola Cerchiello

**Affiliations:** Department of Economics and Management Science, University of Pavia, Pavia, Italy

**Keywords:** ICOs, cryptocurrencies, fundraising, classification models, text analysis, scam

## Abstract

Initial coin offerings (ICOs) are one of the several by-products in the world of the cryptocurrencies. Start-ups and existing businesses are turning to alternative sources of capital as opposed to classical channels like banks or venture capitalists. They can offer the inner value of their business by selling “tokens,” i.e., units of the chosen cryptocurrency, like a regular firm would do by means of an IPO. The investors, of course, hope for an increase in the value of the token in the short term, provided a solid and valid business idea typically described by the ICO issuers in a white paper. However, fraudulent activities perpetrated by unscrupulous actors are frequent and it would be crucial to highlight in advance clear signs of illegal money raising. In this paper, we employ statistical approaches to detect what characteristics of ICOs are significantly related to fraudulent behavior. We leverage a number of different variables like: entrepreneurial skills, Telegram chats, and relative sentiment for each ICO, type of business, issuing country, team characteristics. Through logistic regression, multinomial logistic regression, and text analysis, we are able to shed light on the riskiest ICOs.

## 1. Introduction

Initial Coin Offerings (ICOs) can be considered as an innovative way of obtaining funding, promoted by entrepreneurial companies that base their business projects on a new technology known as blockchain. Up to the present date, more than 1,700 cryptocurrencies have been created but not all of them are successful or characterized by a significant impact. ICOs issue “tokens,” i.e., the unit of a chosen cryptocurrency, in exchange of a flat cryptocurrency, in order to participate in the crowd-funding of the company. Tokens can be bought directly on the web platform of the company, at different stages of the ICO commonly referred as pre-sale and sale. Later, the amount of bought tokens can be sold or used in the future to obtain products or services. The portal Tokendata.io has estimated that until 2017 ICOs raised as much as $5.3 billion around the world; if we consider venture capitalist, in 2016, they invested $71.8 billion in the United States and $4.3 billion in Europe (National Venture Capital Association and Invest Europe). Start-ups and existing businesses are turning to alternative sources of capital as opposed to classical channels like banks or venture capitalists. They can offer the inner value of their business by selling “tokens,” i.e., units of the chosen cryptocurrency, like a regular firm would do by means of an Initial Public Offering (IPO). When we say cryptocurrency, we refer to a digital currency, a new means of exchange, the most popular examples of which are Bitcoin and Ethereum. Blockchain (chain of blocks) is the technology at the basis of a cryptocurrency; it is a Distributed Ledger Technology defined as a distributed, shared, encrypted database that serves as an irreversible and incorruptible repository of information (Wright and De Filippi, 2015). Bitcoin is currently the largest blockchain network followed by, Ethereum, XRP, Litecoin, EOS and Bitcoin Cash (Coinmarketcap, 2018). ICOs favor open-source project development and decentralized business, generating a built-in customer base and positive network effects. They also create a secondary market where tokens can be employed as rewards for using the app of the company or the offered services (Subramanian, 2018). This work aims at addressing the specific characteristics of ICOs using relevant variables that play a key role in determining the success of the ICO.

As it stands there is no database with the information we are looking for, thus we have been building and constantly maintaining a dataset that is currently composed of 196 ICOs that occurred between October 2017 and November 2018 (Cerchiello et al., 2019). The database comprises companies from European countries namely France, Germany, Switzerland, Estonia, Latvia, and non European countries such as Russia, United Kingdom, United States, Japan, Singapore, and Australia. The most common sectors in which ICOs operate are: high-tech services, financial services, smart contract, gambling platforms, marketplaces, and exchanges.

## 2. Initial Coin Offerings

Most of the ICOs projects are related to the development of a blockchain, the issuance of new cryptocurrencies or somehow related fintech services. ICOs tokens grant contributors the right to access platform services in 68.0% of the cases, governance powers in 24.9% of the cases and profit rights in 26.1% of the cases. The secondary market for ICOs tokens is quite liquid on the first day of trading, and the initial return is large (mean value +919.9% compared to the offer price, median value +24.7%). The success of such decentralized technology lays on the fact that it works without the commitment and the control of a central authority: the blockchain is a Peer-to-Peer technology. A Peer-to-Peer (P2P) system represents a way of structuring distributed applications such that the individual nodes can act as both a client and a server. A key concept for P2P systems is to allow any two peers to communicate with each other in such a way that either ought to be able to initiate the contact (Peer-to-Peer Research Group, 2013). Then, the more a P2P network is distributed, scalable, autonomous, and secure, the more is valuable.

All of these precious features have enabled the fast growth of cryptocurrencies not just *per se* but also as a tool for crow-funding purposes, giving birth to the so-called Initial Coin Offerings. Moreover, what is further fueling the development of ICOs, according to BIS Annual Economic Report (2018) is the absence of regulation (even if many countries are currently working on it) and, at the moment, there are just a few examples of banning acts (namely China, India, South Korea). Investors buy ICO tokens in the hope of very high returns, sometimes even before the business is put in place, since the corresponding cryptocurrencies (typically Ethereum) can be immediately traded. In the first 6 months of 2018, there have been 440 ICOs, with a peak in May (125) raising more than 10 billion US, where Telegram ICO (Pre-sale 1 and 2) is by far the most reworded one with 1.7 billion US (Coinschedule, 2018). In 2017, the total amount raised by 210 ICOs was about 4 billion US and overcame venture capital funneled toward high tech initiatives in the same period. The first token sale was held by Mastercoin in July 2013 but one of the most successful and still operative is Ethereum which raised 3,700 BTC in its first 12 h in 2014, equal to approximately 2.3 million dollars at that time.

Recently, there has been a growing literature studying the ICOs drivers aiming to predict their future outcome. A previous study offers an exploratory empiric classification of ICOs and the dynamics of voluntary disclosures. It examines to what extent the availability and quality of the information disclosed can explain the characteristics of success and failure among ICOs and the corresponding projects (Blaseg, 2018). Another important research focuses on the effectiveness of signaling ventures and ICOs projects technological capabilities to attract higher amounts of funding (Fisch, 2019). Momtaz aims at identifying the likelihood and possible timeframe of value creation for investors by combining several factors (financial return, amount of capital raised, listing, and delisting alternatives, industry events study etc.) to analyse the ICOs success drivers (Momtaz, 2018a).

Other streams of research concentrate on the impact of managers quality on the ICOs. Momtaz studies the impact of CEOs loyalty disposition and the magnitude of asymmetry of information between managers and investors on ICOs performance (Momtaz, 2018b). Moreover, to remain in the management area, an interesting spark comes from a research specifically directed on CEOs role and effects on ICOs results (Momtaz, 2018c). Finally, another area of studies focuses on the driving factors impacting the liquidity and trading volume of crypto tokens listed after the ICOs. Among those factors have been identified the quality level of disclosed documentation (source code public on Github, white paper published, an intended budget published for use of proceeds), the community engagement (measured by the number of Telegram group members), the level of preparation of the management (using as proxy the entrepreneurial professional background of the lead founder or CEO), and other outcomes of interest (i.e., the amount raised in the ICO, outright failure—delisting or disappearance, abnormal returns, and volatility) (Howell et al., 2018).

Despite the interest that has been peaked by ICOs and the constantly growing trends, it is worth mentioning that almost half of ICOs sold in 2017 failed by February 2018 (Hankin, 2018). In fact, what should drive more attention to ICOs is the consistent presence of scam activities only devoted to raising money in a fraudulent way. According to Cointelegraph, the Ethereum network (the prevalent blockchain platform for ICOs) has experienced considerable phishing, Ponzi schemes, and other scam events, accounting for about 10% of ICOs (Ethereumscamdb, 2018). On the other hand, it is interesting to assess what factors affect the probability of success of an ICO. Adhami et al. (2017), based on the analysis of 253 ICOs, showed that the following characteristics contribute: the availability of the code source, the organization of a token presale and the possibility for contributors to access to a specific service (or to share profits).

The boom of the ICOs projects and their interesting characteristic brought an important rise of interest from the general audience, many scientific studies have been conducted and published in the last years. Besides the aforementioned Adhami et al. (2017), we should mention the working paper by Zetzsche et al. (2017), that is focused on legal and financial risk aspects of ICOs, moreover a taxonomy is provided and some additional data on ICOs that the authors claim to be continuously updated. Recently, Subramanian in 2018 quoted the ICOs as an example of the decentralized blockchain-based electronic marketplace. The main source of information about blockchain, tokens, and ICOs is obviously the Web. Here we can find sites enabling to explore the various blockchains associated to the main cryptocurrencies, including Ethereum's one. We can also find websites giving extensive financial information on prices of all the main cryptocurrencies and tokens, sites specialized in listing the existing ICOs and giving information about them. Often, these sites evaluate the soundness and likeliness of success of the listed ICOs. One of the most popular among these sites is icobench.com, which evaluates all the listed ICOs and provides an API (Application Programming Interface) to automatically gather information on them. ICOs are usually characterized by the following features: a business idea, most of the time explained in a white paper, a proposed team, a target sum to be collected, a given number of tokens to be given to subscribers according to a predetermined exchange rate with one or more existing cryptocurrencies. Nowadays, a high percentage of ICOs are managed through Smart Contracts running on Ethereum blockchain, and in particular through ERC-20 Token Standard Contract (Fenu et al., 2018).

On top of all the characteristics explained so far, there is a further and not yet explored point of interest: the Telegram chats. Telegram is a cloud-based instant messaging and voice over IP service developed by Telegram Messenger founded by the Russian entrepreneur Pavel Durov. In March 2018, Telegram stated that it has 200 million monthly active users—“This is an insane number by any standards. If Telegram were a country, it would have been the sixth largest country in the world (Telegram, 2018).” Telegram is completely free and has no ads, users can send any kind of media or documents and can program messages to self-destruct after a certain period of time. Some characteristics are imposing Telegram among the first social networks, indeed it intentionally does not collect data about where its clients live and what they use the platform for. This is one of the main reason why, according to AppAnnie rankings, Telegram is particularly popular in countries like Uzbekistan, Ukraine, and Russia, where Internet access may be limited or closely monitored by the government. As of October 2017, Telegram was by far the most popular official discussion platform for current and upcoming ICOs, with 75%+ of these projects employing it. This means that retrieving Telegram discussions associated with each and every ICO would produce a huge amount of textual information potentially useful for understanding the chance of success and more interestingly possible signs of fraudulent activities.

## 3. Methodology

In this paper we leverage two kinds of information: structured and unstructured ones. Regarding the former, we take advantage of classical statistical classification models to distinguish the stauts of an ICO that made up of 3 classes, intended as follows:

- **Success**: the ICO collects the predefined hard cap within the time horizon of the campaign;
- **Failure**: the ICO does not collect the predefined hard cap within the time horizon of the campaign;
- **Scam**: the ICO is discovered to be a fraudulent activity with malicious intent during the campaign and described as such by all the platforms we use for data gathering (namely ICObench and Telegram). A robustness check for the scam labeling come by checking if regulatory bodies announced legal actions against the issuers (e.g., official SEC announcements of legal infringement).

Logistic regression aims at classifying the dependent variable into two groups, characterized by a different status [1 = scam vs 0 = success or 1 = success vs 0 = failure] according to the following model:

(1) $$\begin{array}{l} ln\left(\frac{p_{i}}{1-p_{i}}\right)=\alpha +\underset{j}{\sum }\beta_{j}x_{ij}, \end{array}$$

where *p*_*i*_ is the probability of the event of interest, for ICO *i*, *x*_*i*_ = (*x*_*i*1_, …, *x*_*ij*_, …, *x*_*iJ*_) is a vector of ICOs-specific explanatory variables, the intercept parameter α, as well as the regression coefficients β_*j*_, for *j* = 1, …, *J*, are to be estimated from the available data. It follows that the probability of success (or scam) can be obtained as:

(2) $$\begin{array}{l} p_{i}=\frac{1}{1+exp-\left(\alpha +\sum_{j}\beta_{j}x_{ij}\right)}, \end{array}$$

Since the target variable is naturally categorized according to three classes, success, failure, and scam we extend the aforementioned binary logistic regression to a multinomial one. Such model assesses all the categories of interest at the same time as follows:

(3) $$\begin{array}{l} ln\left(\frac{p_{k}}{1-p_{K}}\right)=\alpha_{k}+\underset{j}{\sum }\beta_{k}x_{ij}, \end{array}$$

where *p*_*k*_ is the probability of *kth* class for *k* = 1, …, *K* given the constraint that $\sum_{K}p_{k}=1$.

Considering the textual analysis of Telegram chats, we take advantage of quantitative analysis of human languages to discover common features of written text. In particular the analysis of relatively short text messages like those appearing on micro-blogging platform presents a number of challenges. Some of these are, the informal conversation (e.g., slang words, repeated letters, emoticons) and the level of implied knowledge necessary to understand the topics of discussion. Moreover, it is important to consider the high level of noise contained in the chats, witnessed by the fact that only a fraction of them with respect to the total number available is employed in our sentiment analysis.

We have applied a Bag of Word (BoW) approach, according to which a text is represented as an unordered collection of words, considering only their counts in each comment of the chat. The word and document vectorization has been carried out by collecting all the word frequencies in a Term Document Matrix (TDM). Afterwards, such matrix has been weighted by employing the popular TF-IDF (Term Frequency Inverse Document Frequency) algorithm. Classical text cleaning procedures have been put in place like stop-words, punctuation, unnecessary symbols and space removal, specific topic words addition. For descriptive purposes we have used word-clouds for each and every Telegram chat according to the general content and to specific subcategories like sentiments and expressed moods. The most critical part of the analysis relies on the sentiment classification. In general, two different approaches can be used:

- Score dictionary based: the sentiment score is based on the number of matches between predefined list of positive and negative words and terms contained in each text source (a tweet, a sentence, a whole paragraph);
- Score classifier based: a proper statistical classifier is trained on a large enough dataset of pre-labeled examples and then used to predict the sentiment class of a new example.

However, the second option is rarely feasible because in order to fit a good classifier, a huge amount of pre-classified examples is needed and this represents a particularly complicated task when dealing with short and extremely non conventional text like micro-blogging chats (Cerchiello and Nicola, 2018). Insofar, we decided to focus on a dictionary based approach, adapting appropriate lists of positive and negative words relevant to ICOs topics in English language. We employ three vocabularies from the R package “tidytext”:

- AFINN from Finn Årup Nielsen;
- BING from Bing Liu and collaborators;
- NRC from Saif Mohammad and Peter Turney.

These lexicons are based on unigrams, i.e., single words, they contain many English words and the words are labeled with scores for positive/negative sentiment and also possibly emotions like joy, anger, sadness, and so forth. The NRC lexicon categorizes words in a binary fashion (“yes”/“no”) into categories of positive, negative, anger, anticipation, disgust, fear, joy, sadness, surprise, and trust. The BING lexicon categorizes words into a binary manner into positive and negative categories. The AFINN lexicon assigns words with a score that runs between −5 and 5, with negative scores indicating negative sentiment and positive scores indicating positive sentiment. By applying the above described lexicons, we produce for each and every ICO a sentiment score as well as counts for positive and negative words. All these indexes are used as additional predictors within the logistic models.

## 4. Data

In this paper, we examine 196 ICOs starting from January 2017 till November 2018. For each project, we gather information from web-based sources, mainly rating platforms such as icobench.com, TokenData.io, ICO Drops.com, CoinDesk.com and project's websites. The process of building up the ICOs data set reflects the main phases that an ICO follows to be launched: from the birth of the business idea, the team building, the purpose of the tokens, the technical requirements (white paper), the promotion and the execution phase.

### 4.1. Collection of Structured Data

The first step in collecting data about each project is to gather information from the most used ICO related platforms as Icobench, TokenData, Coinschedule, or similar. During such phase, we look for general characteristics such as the name, the token symbol, start, and end dates of the crowdfunding, the country of origin, financial data such as the total number of issued token, the initial price of the token, the platform used, data on the team proposing the ICO, data on the advisory board, data on the availability of the website, availability of white paper and social channels.

Some of these data, such as short and long description, and milestones are textual descriptions. Others are categorical variables, such as the country, the platform, and variables related to the team members (name, role, group). The remaining variables are numeric, with different degrees of discretization. Unfortunately, not all ICOs record all variables, so there are several missing data. The ICO web databases that we use are fully checked in order to minimize the missing values of one of the platforms, therefore we validate the information checking for the details on the website and on the white paper. As a result, the complete set of reliable information comes from the matching between the website and the white paper.

The variables set, continuous and categorical data, show us that the main area of origin of the projects is Europe with the highest percentage in Switzerland and Germany. The Switzerland peak is due to the national regulator approach—FINMA (the Swiss Financial Market Supervisory Authority)—which on 16 February 2018 issued clear guidance on the status of ICOs. FINMA does not categorize payment or utility tokens (provided they are not used for investment) as securities. All other tokens are categorized as securities and are subject to securities regulation. To legally issue an equity/asset token, authorization from FINMA should be sought, and appropriate compliance measures [know your customers (KYC) and anti-money laundering (AML)] must be taken. If a debt token can be classified as a deposit, then unless specific exceptions apply, a banking license is needed prior to the ICO. In the fragmented regulatory framework, this is one of the so-called “crypto-friendly” countries, that attract worldwide investors.

The presence of a team of experts as a figure of “advisors” that follows the stages of development are helpful in qualifying the ICO as more reliable. On the development of the dataset the research focused also on assessing the number of advisors for each ICO, checking their educational background and marking as a variable of interest the presence of a Ph.D. that attests a high degree of education.

The evolution of the classic Business Plan that we observe when we analyse the idea of a start-up, is called White Paper. The business plan is the document that illustrates the strategic intentions and the management of competitive strategies of the company, the evolution of key value drivers and the economic and financial results. The drawing up of the operational plan has the aim of achieving different subjects involved in the business. The content of the business plan should not be overlooked, it must be the most possible schematic and of intuitive interpretation. The feature that distinguishes a good plan is the clarity, the synthesis and the professional description of the project workflow. The WP (white paper) therefore fulfills these functions and, in our analysis, played a vital role in the statistical analysis in terms of presence or absence of it. The graphics quality with which it is produced is also important, the data contained within it and the description of the team's components.

### 4.2. Collection of Unstructured Data

Social channels are more personal than every database, rating platform or websites, so they are a way to reach a wide range of users, to update them constantly about the evolution of the project and in the end to create a trusty environment that can finalize in a successful crowdfunding activity. In order to conduct the textual analysis, we enrich our database with the social channels data, such as the presence of a channel, the numbers of users as a proxy of the community engagement and as mentioned in the introduction the textual chat, retrieved in reverse until the creation of the chat. The most used social channels are Telegram, Twitter, Facebook, Bitcointlak, Medium, while Linkedin, Reddit, and Slack are not frequently used.

In crowdfunding projects the entrepreneur and the community in which is embedded works as a strong control for the attractiveness of a business. Some studies have investigated the social network community and the entrepreneurial activity finding out that the amount of capital collected in crowdfunding is heavily dependent on the range of social networks the entrepreneurs belong to (Mollick, 2014).

With regards to the entrepreneurial dimension, we investigate the team components, pointing out that the members checked until now are almost 1,000, with a median size of 7 for project. For each team member we checked general information related to the social engagement, looking for the Linkedin channel activity (48% of them do not have an individual page), the numbers of connections, the job position in the project and the academic background. Moreover, the presence of advisors can play a crucial role in ensuring the reliability of an ICO, provided a wise choice of such advisors. The same applies to institutional investors doing due diligence on a potential venture. In collecting our data, we focused on the academic background and the current area of expertise of the declared advisors.

As it concerns the unstructured data, insightful information can be derived by the white papers in terms of quality of the technical report and specific content. A white paper is a summary report that provides detailed information about the project, its originality and the benefits it can give to investors and users, about the technological features, team behind the project, project's background and future plans. Besides all the above information, we collect Telegram chats associated to each ICO (if available) and apply all the text analytic techniques to produce a sentiment based score.

In Table 1 we report the complete list of collected and employed variables.

**Table 1:** Explanatory variables.

class0	f=failed, sc=scam su=success
class1	0=scam, 1=failed+success
class2	0=failed, 1= success
w_site	Website (dummy)
tm	Telegram (dummy)
w_paper	White paper (dummy)
usd	presale price in USD
tw	Twitter (dummy)
fb	Facebook (dummy)
ln	Linkedin (dummy)
yt	Youtube (dummy)
gith	Github (dummy)
slack	Slack (dummy)
reddit	Reddit (dummy)
btalk	Bitcointalk (dummy)
mm	Medium (dummy)
nr_team	Number of Team members (quantitative)
adv	Existence of advisors (dummy)
nr_adv	Number of advisors (quantitative)
project	Official name of the ICO (categorical)
nr_tm	Number of users in Telegram (quantitative)
tot_token	Number of Total Tokens (quantitative)
Pos_Bing	Standardized nr. of positive words for BL list (quantitative)
Neg_Bing	Standardized nr. of negative words for BL list (quantitative)
Sent_Bing	Standardized sentiment for BL list (quantitative)
Pos_NRC	Standardized nr. of positive words for NRC list (quantitative)
Neg_NRC	Standardized nr. of negative words for NRC list (quantitative)
Sent_NRC	Standardized sentiment for NRC list (quantitative)

## 5. Empirical Evidence

In this section we report our main results obtained from classification analysis and textual analysis. In this regard, in Tables 2, **4** we report results respectively for logistic regression on Success/Failure (class 2 variable) and for multilogit regression estimated on failure (f) and scam (sc) compared to success as baseline. Regarding the first model, in Table 2 we report the final configuration after several stepwise selection steps^1^. The reader can see that the only two relevant dummy variables are: the presence of a white paper (Paper_du) and of a Twitter account (tw). Both present positive coefficients showing their impact on increasing the probability of success of an ICO. It should be stressed that the influence of Twitter channel is much higher than the presence of a white paper, indeed if we calculate the associated odds ratio we would get, respectively 11.94 and 3.85. In other words, if the ICO has a Twitter account the probability of success is almost 12 times higher (almost 4 times higher for the white paper). Regarding the three continuous variables, number of elements of the team (Nr_team), number of advisors (Nr_adv), and scaled sentiment score based on NRC lexicon (Sent_NRC_sc), they are all highly significant and again positive suggesting that increasing people and advisors in the team has a positive impact. Regarding the sentiment, we notice a particularly high positive value, stressing the importance of the perception of possible investors which interact with the ICO proposer by means of a social media, namely Telegram.

**Table 2:** Logistic regression results on success/failure ICOs.

	**Dependent variable**
	**Class 2**
tw	2.481^*^
	(1.381)
Paper_du	1.351^**^
	(0.635)
nr_adv	0.461^***^
	(0.135)
nr_team	0.233^***^
	(0.088)
Sent_NRC_sc	2.187^***^
	(0.595)
Constant	−3.601^**^
	(1.458)
Observations	196
Akaike Inf. Crit.	89.41
McFadden pseudo *R*^2^	0.63
McFadden Adj. pseudo *R*^2^	0.57
Cox & Snell pseudo *R*^2^	0.49

* *p < 0.1*;

** *p < 0.05*;

*** *p < 0.01*.

To further evaluate such configuration, we have explored the VIF index that accounts for the level of multicollinearity brought by each and every variable. The VIF results for the two model configurations are reported in Table 3 (logistic) and 5 (multinomial), with useful insights in defining the lack of multicollinearity^2^. Therefore, in Table 3 we can see low values for the VIF index associated to the estimated logistic model (given in Table 2). The reader can easily notice that there is not any multicollinearity effect, making robust the model. Moreover, reported performance indexes, namely AIC and pseudo *R*^2^, present good values above 50%.

**Table 3:** VIF index for logistic regression model.

**tw**	**Paper_du**	**nr_adv**	**nr_team**	**Sent_NRC_sc**
1.229	1.033	1.067	1.053	1.228

In Table 4, we report results for fraudulent and scam ICOs compared to successful ones, on the basis of a multilogit regression. Looking at the estimated parameters, we can infer that the patterns are different. The presence of a website has a positive impact on the probability of being a successful ICO and not a scam. In other words, the absence of this characteristic is a driver of scam activity suspects. Instead the website does not differentiate successful from failures ones. With regards to the presence of advisors and of a white paper, both the variables are significant in differentiating fraudulent from successful ICO, confirming results of logistic regression. No statistical significance for fraudulent ICOs. Lastly, variable on the sentiment score is relevant and with negative sign for both the classes, in other words an increasing in the sentiment causes an increasing in the probability of success when we consider both failed and fraudulent ICOs.

**Table 4:** Results from multilogit regression: failure and scam compared to success.

	**Dependent variable**	
	**sc**	**f**
	**(1)**	**(2)**
Oweb_dum	−1.962^**^	0.093
	(0.977)	(0.773)
adv_dum	−0.899	−1.707^***^
	(0.809)	(0.571)
Paper_du	−0.728	−2.158^***^
	(0.915)	(0.657)
Sent_NRC_sc	−1.390^*^	−2.606^***^
	(0.731)	(0.703)
Constant	−0.628	−0.572
	(0.997)	(0.925)
Akaike Inf. Crit.	166.339	166.339
*Pseudo R square*	McFadden 0.43 - McFadden Adj. 0.36- Cox & Snell 0.44	

* *p < 0.1*;

** *p < 0.05*;

*** *p < 0.01*.

In this regard, we should stress that the incidence of scam ICOs in our database is extremely low, this due to the fact that collecting information about such ICOs is particularly complex. Most of the information is completely deleted from the Web as soon as the activity is recognized as illegal and/or fraudulent. The overall model performance, assessed again in terms of AIC and pseudo *R*^2^, is pretty good although inferior to the previous one.

In Table 5, we also report VIF index, so to check the absence of multicollinearity in the reported model. Please note that, multilogit model reported in Table 4 is a final configuration obtained through stepwise selection. The full models are available in the Appendix (Supplementary Material)^3^.

**Table 5:** VIF index for multilogit regression model.

**Oweb_dum**	**adv_dum**	**Paper_du**	**Sent_NRC_sc**
3.656	2.317	3.607	3.870

## 6. Discussion and Conclusions

Initial coin offerings (ICOs) are one of the several by-products of the cryptocurrencies world. IPOStart-ups and existing businesses are turning to alternative sources of capital as opposed to classical channels like banks or venture capitalists. They can offer the inner value of their business by selling “tokens,” i.e., units of the chosen cryptocurrency, like a regular firm would do by means of an. The investors, of course, hope for an increase in the value of the token in the short term, provided a solid and valid business idea typically described by the ICO issuers in a white paper. However, fraudulent activities perpetrated by unscrupulous actors are frequent and it would be crucial to highlight in advance clear signs of illegal money raising.

In this perspective, ICOs analysis can be considered a very particular type of fraud detection activity. However, in our opinion fraud detection presents some specificity that prevent us from entailing ICOs related problems as a proper instance of fraud detection. In particular, our data are not flowing in such huge amount from an on-line system as typically happens with credit card payments or banks transactions. Typical fraud detection approaches, as in Maheshwara Reddy et al. (2019), aim at discovering, almost in real times, fraudulent financial activities based on transactional data that ideally should be blocked as soon as possible. ICOs instead are characterized by a slow process of engagement of the prospect clients and establishment of consensus that goes through Telegram chats (if available), white paper and website. That being the case, we would suggest to label this specific stream of research as FinTech Fraud detection with all the relative specificity.

While analyzing success vs failure dynamic with a classification model is relatively easy since the incidence of the two classes is almost equal (50–50), it is much more complicated to highlight the key aspects that could witness a fraudulent activity since, in the last 3 years, only few scam events have been reported. In our sample made of 196 ICOs (data collection still active) we have 10 scam ICOs and we fit a multilogit regression model for comparing scam and failed ICOs toward successful ones. Results tell us that the presence of a website has a positive impact on the probability of not being a scam but does not have any impact on failed ones. In terms of sentiment expressed on Telegram chats, the impact appears to be negative both on the scam and failed ICOs. This suggests that monitoring in real time Telegram chats could represent a valid mean for collecting signs of possible problems within the ICOs. If instead, we compare Successful ICOs against Failed ones, we find that the presence of a White Paper and of a Twitter account show positive coefficients.

Regarding the three continuous variables, number of elements of the team, number of advisors, and sentiment score based on NRC lexicon, they are all highly significant and positive suggesting that increasing people in the team and advisors has a positive impact. Regarding the sentiment, we notice a particularly high positive value, stressing the importance of the perception of possible investors which interact with the ICO proposer by means of a social media.

The paper will be improved in the future by increasing the size of the sample and exploring alternative approaches for textual analysis with specific attention to sentiment analysis. We aim at producing a more refined and tailored sentiment score for each ICO, improving and increasing the vocabulary of words. Specifically regarding the textual analysis an alternative approach that we could use is the combination of words as in Bolasco and Pavone (2017).

As a final remark, authors are aware of the limits of the paper mainly due to the size of the sample. However, given the still limited literature in this field with no reference to the power of textual information collectable through Telegram chats, this contribution represents a step ahead in the process of understanding the ICOs phenomenon. Furthermore a different approach would be to study the trends of the ICOs by combining the available information from specialized websites on fraudulent activities (such as cyphertrace.com and deadcoin.com) and rating websites for the active projects.

## Data Availability Statement

The datasets generated for this study are available on request to the corresponding author.

## Author Contributions

The paper is the product of full collaboration among the authors, however PC has inspired the idea, the methodology and wrote sections 1, 3 and 6, AT run the analysis and wrote sections Keywords, 2, 4 and 5.

### Conflict of Interest

The authors declare that the research was conducted in the absence of any commercial or financial relationships that could be construed as a potential conflict of interest.

## Footnotes

^1^The full model is available in Table A1 in the appendix and it evidently contains several not significant variables.

^2^In Table A2, we have VIF index obtained from the full model and there are high values for some variables, specifically those related to sentiment analysis.

^3^Full multilogit regression model is available in Table A3 in the appendix and in Table A4 the associated VIF index table.
