# Supplementary material for: Initial Coin Offerings: Risk or Opportunity?
Source: Front Artif Intell. 2020 Apr 16;3:18. doi: 10.3389/frai.2020.00018 (PMC7861230; doi:10.3389/frai.2020.00018)
Supplement: Supplementary file 1 [file Data_Sheet_1.pdf]

APPENDIX

Table 1 Logistic regression full model

Variables	*Dependent variable:* Coeff.& s.e.
	class2
Oweb_dum	0.096
	(1.155)
Code_dum	−0.416
	(0.923)
Telegram_du	−0.492
	(1.073)
tw	2.858
	(1.773)
fb	0.107
	(0.817)
ln	−1.236
	(0.927)
yt	1.495
	(1.002)
gith	−0.432
	(0.865)
slack	−0.915
	(1.105)
reddit	−0.067
	(0.902)
btalk	−0.611
	(0.811)
ew	10.598
	(1,648.456)
mm	0.997
	(0.902)
Neg_Bing_sc	−5.293
	(3.846)
Pos_Bing_sc	2.730
	(1.697)
Neg_NRC_sc	4.454
	(4.058)
Pos_NRC_sc	0.749
	(1.829)
Paper_du	1.487
	(1.028)
nr_adv	0.582***
	(0.197)
nr_team	0.268**
	(0.124)
Constant	−3.649*
	(2.018)

Continuation of Table 1	
**Variables**	**Coeff.& s.e.**
**Observations**	**196**
**Log Likelihood**	**−31.727**
**Akaike Inf. Crit.**	**105.454**
Note:	*p<0.1; **p<0.05; ***p<0.01

Table 2. VIF index for the full model

Oweb_dum	Code_dum	Telegram_du	tw	fb
2.034	1.394	2.499	1.572	1.419
ln	yt	gith	slack	reddit
2.058	1.649	1.805	1.348	2.009
btalk	ew	mm	Neg_Bing_sc	Pos_Bing_sc
1.585	1.000	2.003	73.564	9.982
Neg_NRC_sc	Pos_NRC_sc	Paper_du	nr_adv	nr_team
70.371	12.830	2.255	1.703	1.809

Table 3 Multilogit regression full model

	Dependent variable:	
**Variables**	**Coeff.& s.e.**	
	**sc**	**f**
	**(1)**	**(2)**
**Oweb_dum**	−4.568**	1.602
	(2.261)	(1.613)
**Code_dum**	−1.051	1.312
	(1.745)	(1.222)
**Telegram_du**	3.146	0.275
	(2.809)	(1.340)
**tw**	−8.198**	−5.940
	(4.160)	(3.974)
**fb**	3.333	−0.273
	(2.577)	(0.933)
**ln**	1.631	1.448
	(1.945)	(1.185)
**yt**	−2.427	−1.007
	(2.494)	(1.241)
**gith**	0.693	−0.044
	(1.713)	(1.057)
**slack**	−0.834	1.616
	(2.469)	(1.351)
**reddit**	2.058	−0.551
	(2.338)	(1.133)
**btalk**	0.641	0.774
	(1.713)	(0.927)
**ew**	−0.849	−2.641
	(137.854)	(34.907)

Continuation of Table 3		
Variables	Coeff.& s.e.	
	sc	f
	(1)	(2)
mm	−1.703	−0.229
	(2.317)	(1.214)
Neg_Bing_sc	15.599	−6.986
	(13.027)	(8.735)
Pos_Bing_sc	−4.379	−10.411
	(2.882)	(12.921)
Neg_NRC_sc	−14.856	5.266
	(15.289)	(5.423)
Pos_NRC_sc	−1.593	6.095
	(3.984)	(8.983)
Paper_du	−1.457	−2.143*
	(1.744)	(1.243)
nr_adv	−0.564	−0.715***
	(0.377)	(0.254)
nr_team	−0.047	−0.454***
	(0.106)	(0.148)
Constant	0.369	4.372
	(4.177)	(3.062)
Akaike Inf. Crit.	163.240	163.240
Pseudo R square	McFadden 0.46 - McFadden Adj. 0.37 - Cox & Snell 0.46	
Note:	*p<0.1; **p<0.05; ***p<0.01	

Table 4. VIF index multionomial full model

Oweb_dum	Code_dum	Telegram_du	tw	fb
15.583	3.165	9.422	126.167	5.164
ln	yt	gith	slack	reddit
3.883	2.276	4.194	2.408	4.640
btalk	ew	mm	Neg_Bing_sc	Pos_Bing_sc
3.443	1.007	6.435	575.279	1,293.780
Neg_NRC_sc	Pos_NRC_sc	Paper_du	nr_adv	nr_team
241.850	770.032	7.955	3.908	8.418
